# Cichlid Fishes in the Angolan Headwaters Region: Molecular Evidence of the Ichthyofaunal Contact between the Cuanza and Okavango-Zambezi Systems

**DOI:** 10.1371/journal.pone.0065047

**Published:** 2013-05-27

**Authors:** Zuzana Musilová, Lukáš Kalous, Miloslav Petrtýl, Petra Chaloupková

**Affiliations:** 1 Laboratory of Fish Genetics, Institute of Animal Physiology and Genetics AV ČR v.v.i, Liběchov, Czech Republic; 2 Department of Zoology and Fisheries, Faculty of Agrobiology, Food and Natural Resources, Czech University of Life Sciences Prague, Prague, Czech Republic; 3 Department of Zoology, Faculty of Science, Charles University in Prague, Prague, Czech Republic; 4 Institute of Tropics and Subtropics, Czech University of Life Sciences Prague, Prague, Czech Republic; University of Bologna, Italy

## Abstract

The headwaters of five large African river basins flow through the Bié Plateau in Angola and still remain faunistically largely unexplored. We investigated fish fauna from the Cuanza and Okavango-Zambezi river systems from central Angola. We reconstructed molecular phylogenies of the most common cichlid species from the region, *Tilapia sparrmanii* and *Serranochromis macrocephalus*, using both mitochondrial and nuclear markers. We found evidence for ichthyofaunal contact and gene flow between the Cuanza and Okavango-Zambezi watersheds in the Bié Plateau in central Angola. Waterfalls and rapids also appeared to restrict genetic exchange among populations within the Cuanza basin. Further, we found that the Angolan Serranochromis cichlid fishes represent a monophyletic lineage with respect to other haplochromines, including the serranochromines from the Congo and Zambezi rivers. This study represents an important initial step in a biodiversity survey of this extremely poorly explored region, as well as giving further understanding to species distributions and gene flow both between and within river basins.

## Introduction

Natural geographical barriers separating watersheds, such as mountain ranges that divide river basins (“inter-basin barriers”), dictate the distributions of freshwater organisms. Similarly, barriers within the rivers, such as waterfalls and rapids zones can influence species distribution patterns (“intra-basin barriers”) [1], [2], [3]. Such barriers can limit the gene flow and even initiate speciation [4], [5], [6], [7]. In conjunction with inter- and intra-basin barriers, geological history can strongly impact distribution patterns of freshwater ichthyofauna. During geological events (such as the post-Miocene plate tectonics in case of Africa [8], [9]), river systems can be reshuffled by river capture, resulting in many river systems sharing a significant portion of their fauna [10]. African rivers are generally characterized by high frequencies of rapids and waterfalls, when compared to the rest of the world’s rivers [10].

Despite the numerous geographic barriers, African rivers are characterized by high levels of connectivity, possessing species with very broad distribution ranges throughout the continent [11]. Several studies have found semipermeable watersheds, which indicate the presence of weak barriers between river systems, leading to incomplete faunal isolation with persistence of the gene flow [11], [12], [13], [14].

**Figure 1:**
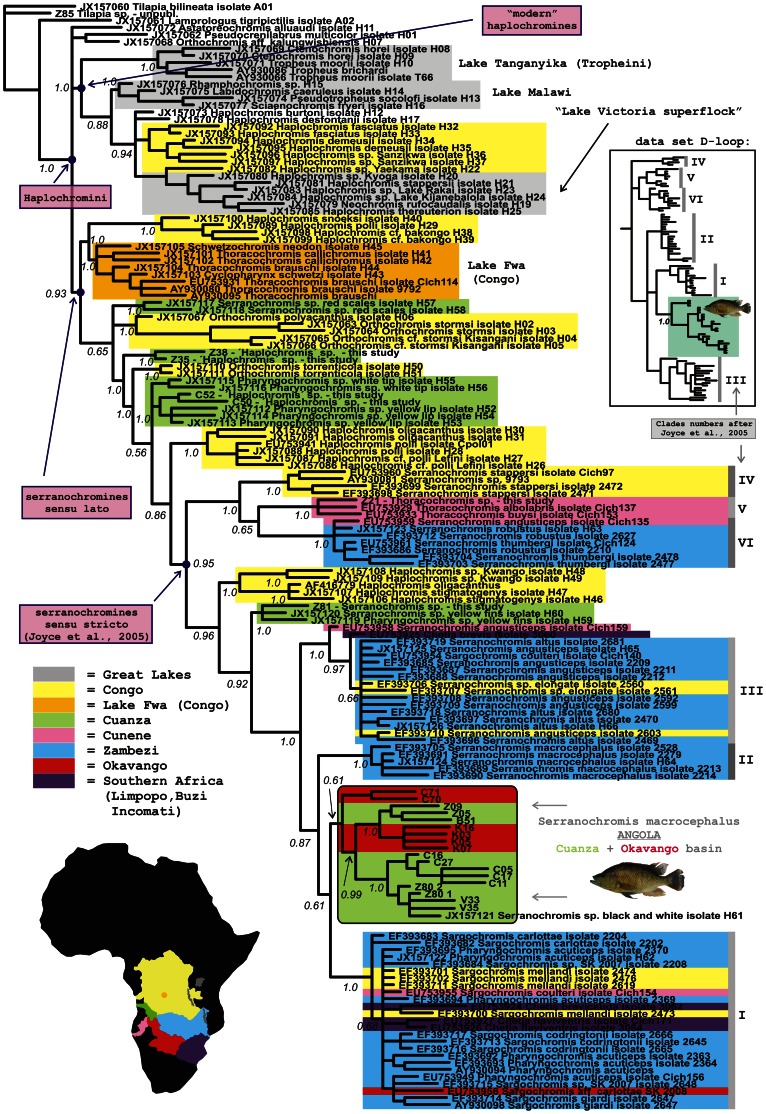
Phylogenetic tree of haplochromine cichlids focused on the serranochromines. Available sequences from previous studies in combination with newly collected specimens were used for the analysis. GenBank accession number mentioned for each sample. Bayesian tree based on the sequence data of mitochondrial ND2 gene. (D-loop based tree shown in the separate schematic cut-out).

Cichlid fishes, together with cyprinids and catfishes, are the dominant part of the African freshwater ichthyofauna in terms of both species diversity and abundance. In southern Africa, three cichlid groups, hemichromines, haplochromines and tilapiines, represent the majority of their biodiversity [15]. Tilapiines in Angola are represented by several phylogenetic lineages [16], [17] and the genus *Tilapia* from the tilapiine group is characterized by omnivorous substrate spawning reproduction srtategy. Haplochromine cichlids consist of a few basal forms (genus *Pseudocrenilabrus*, some ‘*Orthochromis’* species) and two other large species-rich lineages. The first lineage, also known as “modern haplochromines” is represented mostly by lacustrine forms to which the hundreds of the most famous East African lakes cichlids belong [18]. The second lineage is represented by mainly riverine haplochromines corresponding to the name “*Serranochromines sensu lato*” ([19]; see also Fig. 1). Fishes of the genus *Serranochromis* then belong to the subgroup of this “riverine haplochromine” lineage, called “*Serranochromines sensu stricto*” [13]. All *Serranochromis* species are predators with a mouthbrooding reproduction strategy.

Whilst little is known about the biogeography of the genus *Tilapia*, the origin and distribution patterns of serranochromine cichlids are better understood [13]. Studies conducted mainly on cichlids from Eastern and Southern Africa proposed the origin of serranochromine (*sensu stricto*) cichlids within the Kalahari Paleolakes area (formerly Paleo-Makgadikgadi Lake; [13], [20], [21]), which dried out only in the Holocene [21]. This paleobasin was located in what is now the upper/middle Zambezi and Okavango delta region, surrounded by the Kalahari Desert, in southern Zambia and northern Botswana [13]. It is proposed that cichlid fishes underwent a radiation event within this paleolake environment, similar to the radiations observed in the Great African Lakes. The radiation would have been followed by waves of colonization to the riverine habitats, leading mainly to the faunistic enrichment of the Zambezi and Congo basins [13]. Consequently, the frequent repetitive bottlenecks decreasing the intrapopulation variation, followed by genetic drift and repeated (re)colonizations, hybridization and introgression, may have played an important role in the evolutionary history of the serranochromine cichlids. Using genetic data, specific “mosaic” distribution patterns have been repeatedly observed in the region, including the sharing of haplotypes in geographically distant species, as well as the presence of divergent haplotypes within the particular populations [13], [14], [21].

Within this study, we investigate the biogeographical distribution of two cichlid species, the tilapiine *Tilapia sparrmanii*, and the serranochromine *Serranochromis macrocephalus*, focusing within the Bié Plateau in central Angola.

The Bié Plateau in central Angola represents a headwaters region with tributaries of the five large important African river systems (the Congo, Zambezi, Okavango, Cuanza and Cunene). All these rivers tributaries are situated in a relatively small area of about 15,000 km^2^. The Cuanza (Kwanza) River is one of the westward flowing streams in Angola with its basin covering a narrow coastal plain and a steep escarpment rising to an altitude of more than 1,500 m above sea level [22]. The lower and middle parts of the Cuanza river, together with other smaller coastal rivers, represent a distinct Freshwater Ecoregion [23], while the upper Cuanza river is considered as a part of the Zambezian Headwaters Ecoregion based on species relatedness to the Zambezi and Cunene basins [23], [24]. This distinction is mainly supported by a few historic faunistic records [25], [26]. The Okavango is the river system with headwaters in Angola and Namibia, flowing south-eastwards, and emptying through the inland continental delta in Botswana located in the recent Kalahari desert, but which used to be part of the aforementioned Kalahari Paleolakes; [13], [20]. This Paleolake used to largely connect the Okavango and Zambezi river systems in past; however a temporary narrow connection still persists between the Okavango delta and middle Zambezi during high water periods [15].

The Bié Plateau in central Angola represents also one of the least faunistically explored regions in Africa [15], [27] with only a handful of historical studies on ichthyofauna [24], [25], [28]–[33]. Ichthyological research in central Angola since 1975 has been largely prevented by a long-lasting civil war, and the persistent danger of landmines in many areas.

**Figure 2:**
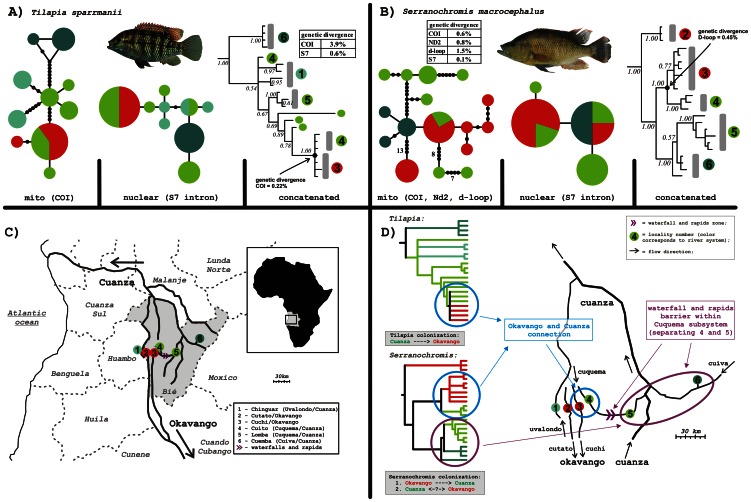
Molecular phylogeny of two fish species from central Angola suggests connection of ichthyofauna between the two river systems. Cladograms and haplotype networks for two cichlid species, A) *Tilapia sparrmanii* (CO1 and S7 intron) and B) *Serranochromis macrocephalus* (CO1, D-loop, ND2 and S7 intron). Trees represent results of the Bayesian analyses in MrBayes run for 5 million generations (burn-in 25%). Haplotype networks are based on mitochondrial gene(s) and nuclear gene (S7 intro). C) Sampling localities in the Bié province (highlighted in grey) in the central Angola with the river systems scheme. Some of the localities could be represented by more collection sites. D) Schematic detailed map and schematic cladograms colored by the river system. Presence of the connection between two river systems enabled the putative colonization events. Light blue-green (no. 1)  =  the Uvalondo subsystem (the Cuanza system), green (4, 5)  =  the Cuquema subsystem (the Cuanza system), dark green (6)  =  the Cuiva subsystem (the Cuanza system), and red (2, 3)  =  the Cutato and Cuchi subsystems (the Okavango system). Connection of the ichthyofauna between the Cuanza (Cuquema) and the Okavango river systems found in both cichlid species. Clades showing this connection are highlighted by blue color. Further, in *Serranochromis macrocephalus* possible effect of the waterfall and rapids zone was hypothesized (highlighted by violet) separating localities 4 and 5 in the Cuquema subsystem (the Cuanza system).

Within this study, we focus on two basins in central Angola: 1) the Cuanza river system, and 2) the Okavango river system (also called Okavango – Zambezi because of the persistent temporary connection; [15]). Several small and middle-sized rivers, some of which have been only recently separated by vast flat grassy marshlands, represent the headwaters regions of these basins. The Cuanza river system geographically occupies the overwhelming part of the Bié Plateau, with its tributaries flowing both northward and southward, before converging and turning to the north. In this study we investigate three of the Cuanza river subsystems: the Uvalondo River, the Cuquema River and the Cuiva River (named in the western-eastern order). The Okavango River system also possesses several tributaries situated in the Bié Plateau. In our study, we address two of them, the Cuchi River and the Cutato dos Ganguelas River (Cutato for short), both of which flow southward, forming the frontiers of the Bié and Huambo provinces (Figure 2C, D).

The Bié Plateau provides an excellent system for assessing the impact of inter- and intra-basin barriers on faunal distributions. The distances between the streams of the Okavango and Cuanza systems vary between 15 km and 100 km (Fig. 2 c, d), and contact between the river basins has been postulated, but has never been confirmed using a molecular approach. Finally, in the middle part of the Cuquema River (Cuanza basin), there is a waterfall and several rapids zones, representing another potential physical barrier for fish movement. Very few studies concerning Angolan fishes have been published [20], [34], [35]. Only one study, Schwarzer et al. [20], has included few samples of Angolan cichlids in a phylogenetic study.

Here, we present the first results of molecular analyses testing for connectivity between the Cuanza and Okavango river systems in the Bié Plateau, using the cichlid fishes *Tilapia sparrmanii* and *Serranochromis macrocephalus* as representative species. We first compare locally sampled fish to their broader species distribution across Africa. We then look within fish of the two river basins and test for historic migration between catchments. Finally, we use fish sampled on either side of waterfalls and rapids found in the Cuanza River to test for the effects of intra-basin barriers on species distributions.

## Materials and Methods

### Field Sampling

The samples for this study were collected mainly by seine net, rod or hand net in both smaller creeks and larger streams in the headwaters of the Bié Plateau in Angola between 2007 and 2009. Fish were killed with overdose of the anesthetic Phenoxy-2-ethanol diluted in water. All specimens were photographed and fin-clipped for DNA analyses, and selected voucher specimens were fixed in formaldehyde and stored in the collection of the University of Life Sciences, Prague, Czech Republic. Specimens were collected from the two river systems, Okavango (Kubango) and Cuanza (Kwanza or Quanza), in six localities in total (see also Fig. 2C). All collecting sites were chosen mainly with respect to collectors’ safety and accessibility (transport, landmines). Cichlid fishes of the genera *Tilapia* and *Serranochromis* were preferentially chosen due to their presence in most of the sampled localities (see Table 1 and Figure S1 for the samples overview). The collected fishes were sorted and identified according to their morphology and meristic features, and subsequent species identification was based on the available literature [15], [28].

**Table 1:** Sample list with localities.

Sample No.			locality				
	species	collection date	Map	name	subsystem	system	GPS
C70	*Serranochromis macrocephalus*	30.10.2007	2	mainroad Cuito-Huambo	Rio Cutato	Okavango	S 12 34 13.6 E 016 29 30.5
C71	*Serranochromis macrocephalus*	30.10.2007	2	mainroad Cuito-Huambo	Rio Cutato	Okavango	S 12 34 13.6 E 016 29 30.5
C74	*Serranochromis macrocephalus*	30.10.2007	2	mainroad Cuito-Huambo	Rio Cutato	Okavango	S 12 34 13.6 E 016 29 30.5
K01	*Serranochromis macrocephalus*	1.11.2007	3	mainroad Cuito-Huambo	Rio Cuchi	Okavango	S12 31 52.2 E 016 41 46.1
K03	*Serranochromis macrocephalus*	1.11.2007	3	mainroad Cuito-Huambo	Rio Cuchi	Okavango	S12 31 52.2 E 016 41 46.1
K05	*Serranochromis macrocephalus*	1.11.2007	3	mainroad Cuito-Huambo	Rio Cuchi	Okavango	S12 31 52.2 E 016 41 46.1
K07	*Serranochromis macrocephalus*	1.11.2007	3	mainroad Cuito-Huambo	Rio Cuchi	Okavango	S12 31 52.2 E 016 41 46.1
K16	*Serranochromis macrocephalus*	1.11.2007	3	mainroad Cuito-Huambo	Rio Cuchi	Okavango	S12 31 52.2 E 016 41 46.1
K21	*Serranochromis macrocephalus*	1.11.2007	3	mainroad Cuito-Huambo	Rio Cuchi	Okavango	S12 31 52.2 E 016 41 46.1
K28	*Serranochromis macrocephalus*	1.11.2007	3	mainroad Cuito-Huambo	Rio Cuchi	Okavango	S12 31 52.2 E 016 41 46.1
B51	*Serranochromis macrocephalus*	22.10.2007	4	Cuito env.	Rio Cuquema	Cuanza	S12 28 14.9 E16 49 26.7
Z05	*Serranochromis macrocephalus*	16.10.2008	4	Cuito env.	Rio Cuquema	Cuanza	S12 28 14.9 E16 49 26.7
Z09	*Serranochromis macrocephalus*	17.10.2008	4	Cuito env.	Rio Cuquema	Cuanza	S12 28 14.9 E16 49 26.7
C05	*Serranochromis macrocephalus*	25.10.2007	5	Lomba village	Rio Cuquema	Cuanza	S12 30 55.2 E017 25 58
C11	*Serranochromis macrocephalus*	25.10.2007	5	Lomba village	Rio Cuquema	Cuanza	S12 30 55.2 E017 25 58
C16	*Serranochromis macrocephalus*	25.10.2007	5	Lomba village	Rio Cuquema	Cuanza	S12 30 55.2 E017 25 58
C17	*Serranochromis macrocephalus*	25.10.2007	5	Lomba village	Rio Cuquema	Cuanza	S12 30 55.2 E017 25 58
C27	*Serranochromis macrocephalus*	25.10.2007	5	Lomba village	Rio Cuquema	Cuanza	S12 30 55.2 E017 25 58
C31	*Serranochromis macrocephalus*	25.10.2007	5	Lomba village	Rio Cuquema	Cuanza	S12 30 55.2 E017 25 58
V33	*Serranochromis macrocephalus*	6.5.2008	6	Cuemba village	Rio Cuiva	Cuanza	S12 09 32.1 E18 05 48.7
V35	*Serranochromis macrocephalus*	6.5.2008	6	Cuemba village	Rio Cuiva	Cuanza	S12 09 32.1 E18 05 48.7
Z80_1	*Serranochromis macrocephalus*	february 2009	6	Cuemba village	Rio Cuiva	Cuanza	S12 09 32.1 E18 05 48.7
Z80_2	*Serranochromis macrocephalus*	february 2009	6	Cuemba village	Rio Cuiva	Cuanza	S12 09 32.1 E18 05 48.7
K09	*Tilapia sparrmanii*	1.11.2007	3	mainroad Cuito-Huambo	Rio Cuchi	Okavango	S12 31 52.2 E 016 41 46.1
K10	*Tilapia sparrmanii*	1.11.2007	3	mainroad Cuito-Huambo	Rio Cuchi	Okavango	S12 31 52.2 E 016 41 46.1
K17	*Tilapia sparrmanii*	1.11.2007	3	mainroad Cuito-Huambo	Rio Cuchi	Okavango	S12 31 52.2 E 016 41 46.1
K18	*Tilapia sparrmanii*	1.11.2007	3	mainroad Cuito-Huambo	Rio Cuchi	Okavango	S12 31 52.2 E 016 41 46.1
Z02	*Tilapia sparrmanii*	16.10.2008	4	Cuito env.	Rio Cuquema	Cuanza	S12 26.559 E16 54.385
Z11	*Tilapia sparrmanii*	19.10.2008	1	Chinguar village	Rio Uvalondo	Cuanza	S12 33 26.4 E16 18 45.9
Z12	*Tilapia sparrmanii*	19.10.2008	1	Chinguar village	Rio Uvalondo	Cuanza	S12 33 26.4 E16 18 45.9
Z13	*Tilapia sparrmanii*	19.10.2008	1	Chinguar village	Rio Uvalondo	Cuanza	S12 33 26.4 E16 18 45.9
Z14	*Tilapia sparrmanii*	19.10.2008	1	Chinguar village	Rio Uvalondo	Cuanza	S12 33 26.4 E16 18 45.9
Til8	*Tilapia sparrmanii*	May, 2007	4	Cuito env.	Rio Cuquema	Cuanza	S12 25.542 E16 49.113
K159	*Tilapia sparrmanii*	15.10.2008	5	Lomba village	Rio Cuquema	Cuanza	S12 30 55.2 E017 25 58
K160	*Tilapia sparrmanii*	15.10.2008	5	Lomba village	Rio Cuquema	Cuanza	S12 30 55.2 E017 25 58
Z08	*Tilapia sparrmanii*	16.10.2008	4	Cuito env.	Rio Cuquema	Cuanza	S12 28 14.9 E16 49 26.7
N8	*Tilapia sparrmanii*	25.10.2007	5	Lomba village	Rio Cuquema	Cuanza	S12 30 55.2 E017 25 58
Z10	*Tilapia sparrmanii*	18.10.2008	4	Cuito env.	Rio Cuquema	Cuanza	S12 26.559 E16 54.385
Z51	*Tilapia sparrmanii*	24.10.2008	6	Cuemba village	Rio Cuiva	Cuanza	S12 09 32.1 E18 05 48.7
Z60	*Tilapia sparrmanii*	24.10.2008	6	Cuemba village	Rio Cuiva	Cuanza	S12 09 32.1 E18 05 48.7
Cu01	*Tilapia sparrmanii*	May, 2008	6	Cuemba village	Rio Cuiva	Cuanza	dried specimen from market
Cu02	*Tilapia sparrmanii*	May, 2008	6	Cuemba village	Rio Cuiva	Cuanza	dried specimen from market
Z87	*Tilapia sparrmanii*	October, 2008	4	Cuito village	Rio Cuquema	Cuanza	dried specimen from market
Kw02	*Tilapia sparrmanii*	October, 2008	4	Cuito village	Rio Cuquema	Cuanza	dried specimen from market
Kw06	*Tilapia sparrmanii*	October, 2008	4	Cuito village	Rio Cuquema	Cuanza	dried specimen from market
Z35	* ´Haplochromiś* sp.	22.10.2008	-	Luando waterfall	Rio Luando	Cuanza	S11 35 33.4 E18 28 10.3
Z38	* ´Haplochromiś* sp.	22.10.2008	-	Luando waterfall	Rio Luando	Cuanza	S11 35 33.4 E18 28 10.3
C50	* ´Haplochromiś* sp.	31.10.2007	5	Lomba village	Rio Cuquema	Cuanza	S12 30 55.2 E017 25 58
C52	* ´Haplochromiś* sp.	31.10.2007	5	Lomba village	Rio Cuquema	Cuanza	S12 30 55.2 E017 25 59
Z21	*Thoracochromis* sp.	19.10.2008	-	Huambo env.	Rio Cunene	Cunene	S12 45 40.9 E15 47 22.2
Z81	*Serranochromis* sp.	October, 2008	6	Cuemba village	Rio Cuiva	Cuanza	S12 09 32.1 E18 05 48.7

### DNA analyses

Mitochondrial and nuclear gene segments were amplified for both cichlid species. The mitochondrial COI gene (primers: forward - 5′-TCA ACC AAC CAC AAA GAC ATT GGC AC-3′ and reverse 5′-TAG ACT TCT GGG TGG CCA AAG AAT CA-3′ from [36]) and the nuclear S7 first intron (primers: forward 5′-TGG CCT CTT CCT TGG CCG TC-3′ and reverse 5′-AAC TCG TCT GGC TTT TCG CC-3′ from [37]) marker were used for both species. Additionally the mitochondrial control region (D-loop; primers: forward 5′-CCT ACT CCC AAA GCT AGG ATC-3′ and reverse 5′- TGC GGA GAC TTG CAT GTG TAA G -3′ from [38]) and NADH2 dehydrogenase (primers: forward 5′-CTA CCT GAA GAG ATC AAA A-3′ and reverse 5′-CGC GTT TAG CTG TTA ACT AA-3′ from [39]) were amplified for *Serranochromis macrocephalus*.

DNA was extracted from small pieces of fish fin using the DNeasy^™^ Tissue Kit (Qiagen, Valencia, CA, USA). PCR conditions consisted of an initial denaturation step of 94°C for 2 min, followed by 36 cycles of denaturation at 94°C for 1 min, annealing at 50°C (D-loop), 52°C (CO1), 55°C (ND2) or 59°C (S7 intron) for 1 min and extension at 72°C for 1 min. The terminal extension was at 72°C for 10 min. PCR products were then purified by QIAquick PCR Purification Kit (Qiagen), and sequenced directly using the PCR primers along with the BigDye® Terminator Cycle Sequencing Kit v.1.1 (Applied Biosystems, Foster City, CA, USA) following the manufacturer’s protocol. The sequencing reaction products were cleaned with DyeEx 2.0 Spin Kit (Qiagen), and run on ABI Prism 3130 Genetic Analyzer (Applied Biosystems). A proportion of the samples were sequenced using the Macrogen sequencing service in South Korea (www.macrogen.com). Chromatograms were assembled and checked by eye for potential mistakes, and edited sequences were aligned using Clustal W, as implemented in the BioEdit software package [40]. All obtained sequences were submitted to the GenBank database (Accession Nos. KC146709 - KC146839).

### Phylogenetic analyses

First, in order to evaluate our data within the broader taxonomic and geographical context, we reconstructed a phylogeny using additional available sequence data for the related cichlid species and genera. For the genus *Tilapia* virtually no detailed phylogeographic study exists so far, however for the serranochromine lineage, represented here by the genus *Serranochromis*, has a significantly more sequence data available [13], [20], [21]. This allowed us to conduct the overall phylogenetic analysis combining the data of *Serranochromis macrocephalus* collected here, with the serranochromine (*sensu stricto*) and other (mainly riverine) haplochromine sequence data from previous studies [13], [20], [21], [41]. Two broader phylogenetic analyses are presented herein, first based on the mitochondrial NADH dehydrogenase 2 gene using data from the studies of [20], [21], and second based on the control region (D-loop) data from [13], [20], [41]. Sequences were downloaded from the GenBank database (numbers shown in Fig. 1). Analyses parameters were set as described in the next paragraph.

In our second phylogenetic reconstruction focusing in detail on two river systems, two independent cichlid sequence data sets of *Tilapia sparrmanii* (two genes: COI and S7 first intron) and *Serranochromis macrocephalus* (four genes: COI, ND2, D-loop and S7 first intron) were then analyzed by the Bayesian Inference as implemented in MrBayes 3.0 [42]. The best-fit model for each gene was selected separately by jModeltest [43] using the Bayesian information criterion. The Bayesian analysis was performed using two independent runs of four Metropolis-coupled chains (MCMC) of five million generations each, to estimate the posterior probability distribution. The combined sequence matrices were partitioned per gene fragment, and independent model parameters were estimated for each partition. The tree topologies were sampled every 100 generations, and majority-rule consensus trees were estimated after discarding the first 25% of generations by burn-in. Robustness of the clades was assessed using the Bayesian posterior probabilities.

The phylogenetic trees were rooted by two outgroups for each data set. *Tilapia rendalli* and *Thoracochromis* sp. were used for the *Serranochromis macrocephalus* phylogeny, and *Tilapia rendalli* was used for the *T. sparrmanii* phylogeny.

In addition to the phylogenetic trees, haplotype networks were reconstructed for the mitochondrial and nuclear data sets separately, using TCS software [44]. For *Serranochromis macrocephalus*, three mitochondrial genes were concatenated; for *Tilapia sparrmanii* the only mitochondrial sequenced COI gene was used. The nuclear S7 first intron was analyzed for both species.

### Sequence divergence and estimated dating

We calculated the mean genetic divergence values for the root of phylogenetic tree of each studied species. We also calculated the value for the nodes of the tree, where the Cuanza/Okavango connection occurred. We used PAUP software [45] to obtain the uncorrected p-distances. We then estimated the age of the nodes by dating of the obtained values of genetic divergence for mitochondrial D-loop. For that we applied a range of substitution rates for recent divergence events in cichlids of 0.0324 – 0.057 substitutions per site per million years (MY), which corresponds to the genetic divergence of 6.5 – 11.5% per MY [46]. For *Tilapia sparrmanii* the D-loop data were not available, but the other studied mitochondrial gene (COI) showed a 6,5-fold higher divergence compared to the COI gene of *Serranochromis macrocephalus* (3.9% vs. 0.6% respectively; see Fig 2A, B). We took this ratio as an estimator of the d-loop divergence in *Tilapia* (as this gene was not sequenced in *Tilapia*).

### Tests of alternative topologies

Tests of the alternative topologies for “river-system monophyly” were performed using the Likelihood Ratio Test (LRT) and using the Bayes factor value [47] to statistically evaluate the significance of our results against the hypothetical biogeographic scenario of the two river systems’ separation. We tested observed topology with the topologies constructed under the constraints where: a) all samples collected in the Cuanza system were forced into one clade, b) all samples belonging to the Okavango river system were forced to cluster together, and c) both Cuanza and Okavango samples were constrained separately each into a monophyletic clade. Further, d) all the specimens from Cuquema subsystem (Cuanza) were constrained together. All the phylogenetic analyses (both constrained and unconstrained) were performed in MrBayes under the conditions described above and using the command “constraint”. Values of marginal likelihood were then obtained by the “sump” command in MrBayes for each analysis. LRT works with twice the difference of marginal likelihoods (2×ΔlnL) of constrained vs. unconstrained analyses. The value was then checked for the critical values of chi-square in different levels of significance. Both arithmetic and harmonic means were used for LRT. Similarly, the Bayes factor is defined as twice the difference of harmonic means of marginal likelihoods from MrBayes runs (i.e. again 2×ΔlnL). If this twofold difference is >10, the evidence against the alternative topology is considered as “very strong” [47].

## Results

We sequenced 51 specimens of the most common cichlid fishes (23 specimens of *Serranochromis macrocephalus* and 22 specimens of *Tilapia sparrmanii*, and 6 other haplochromines) from the study areas of the Bié Plateau in central Angola.

In both broader phylogenetic analyses (D-loop and ND2) for haplochromine cichlids incoporating data from previously published studies [13], [20], [21], [41], our samples from central Angola represented a monophyletic group among all the other serranochromines *sensu stricto* (i.e. genera *Serranochromis, Pharyngochromis, Sargochromis, Chetia, Thoracochromis* and few species of * ´Haplochromiś*), see Fig. 1. The conspecific representatives of *Serranochromis macrocephalus* from the Zambezi River were found as closely related, but not monophyletic with the Angolan assemblage. Based on studied markers, the species *Serranochromis macrocephalus* itself has actually been found as a polyphyletic unit (Fig. 1).

The hypothesis of correspondence of the revealed biogeographic pattern to the recent river system separation (i.e., if the populations coming from the same river system cluster together) was rejected since the specimens from the adjacent Cuchi (Okavango) and Cuquema subsystems (Cuanza) represent together one monophyletic lineage in both studied species *Serranochromis macrocephalus,* and *Tilapia sparrmanii* (Fig. 2A, B). This provides strong evidence for the presence of ichthyofaunal connections among the sites from the different river basins.

The age of the species clades studied differed: genetic divergence of the *Serranochromis macrocephalus* clade was 1.5% of uncorrected p-distance for D-loop which corresponds to the age of 0.13 - 0.23 MYA following the molecular clock calibration for D-loop as proposed by Koblmueller et al, [46], i. e., the mutation rate of 0.0324 – 0.057 substitutions/site/MY (6.5 – 11.4% of divergence per MY). The internal clade of *Serranochromis macrocephalus*, where the second colonization event happened, is then 0.039 – 0.069 MY old having genetic divergence in d-loop of 0.45% (see Fig. 2B). The divergence of Angolan *Tilapia sparrmanii* clade was estimated from COI gene giving the artificial value of 9.75% of divergence in D-loop. This corresponds to the age of 0.86 – 1.5 MY for the clade. The internal clade of *Tilapia,* (in which the putative Cuanza-to-Okavango colonization happened), shows a lower genetic divergence of 0.22% in COI gene corresponding to the age of about 0.048 – 0.085 my (considering the artificial estimated D-loop divergence equals to 0.55%).

The four alternative topologies for *Serranochromis* (i.e., Okavango monophyletic, Cuanza monophyletic, both river systems monophyletic and Cuquema monophyletic) and two alternative topologies for *Tilapia* (Cuanza monophyletic, Cuquema monophyletic) were significantly rejected by both the Likelihood Ratio test, and by the Bayes factor in the majority of the cases (Table 2), therefore the clustering pattern according to the river systems is unlikely in both studied species. Similarly, also the populations from the upper and lower Cuquema (Cuanza) were found as well-separated and unlikely to cluster together in all analyses. Figure 2C, D shows the observed distribution pattern of both species in detail.

**Table 2:** Test of alternative topologies of the “river-system monophyly.”

	-lnL from MrBayes					
analysis	harmonic mean	arithmetric mean	LRT significance		Bayes factor (ΔlnL)	
*Serranochromis macrocephalus (*4 genes)						
no constraint	–6406.87	–6441.07				
constraint Cuanza	–6422.67	–6461.24	x	*	15.8	°°°
constraint Okavango	–6442.89	–6482.42	***	***	36.02	°°°
constraint Cua + Oka	–6442.95	–6481.72	***	***	36.08	°°°
constraint Cuquema	–6460.95	–6499.18	***	***	54.08	°°°
*Tilapia sparrmanii* (2 genes)						
no constraint	–2458.58	–2492.36				
constraint Cuanza	–2488.21	–2517.2	***	***	29.63	°°°
constraint Cuquema	–2484.13	–2516.27	***	**	25.55	°°°

The constrained (alternative) topologies with the specimens from the river systems (i. e. Cuquema/Cuanza and/or Okavango) forced to be monophyletic, were tested on both studied cichlid species. Values of –lnL resulted from Bayesian analyses were compared between constrained and unconstrainted topology by the Likelihood Ratio Test (LRT) and the Bayes factor. In most cases the alternative topologies were rejected with the level of significance p = 0.05 (*), 0.01 (**) or 0.001 (***), or a “very strong” evidence against the alternative topologies was provided by the Bayes factor (°°°).

## Discussion

### A. The Angolan cichlids

Our study is a first step in a biodiversity survey in the poorly investigated region of central Angola. Although we identified the studied specimens based on the available literature [15], [28] as representatives of *Serranochromis macrocephalus* and *Tilapia sparrmanii*, this could be questioned as the Angolan cichlids taxonomy is still not fully solved. Based on the broadly studied markers, the mitochondrial ND2 gene and control region (D-loop), the largely distributed species *Serranochromis macrocephalus* itself has been found as a polyphyletic unit, which is in agreement with previous studies [13], [21]. Therefore, some taxonomic changes in *Serranochromis macrocephalus* might happen in future because of these observed phylogenetic inconsistencies within the species. However, we found that all the studied Angolan individuals of *S. macrocephalus* represent a monophyletic lineage within the overall haplochromine phylogenetic tree (see Fig. 1) suggesting the genetic uniqueness of the Angolan lineage. Schwarzer et al. [20] refer to their single upper-Cuanza sample falling also into this Angolan clade as *Serranochromis* sp. “black and white” (see Fig. 1). Until a robust phylogenetic analysis based on genomic data is available, all the taxonomic conclusions are premature. Unfortunately, we have no possibility of broader comparisons for *T. sparrmanii*, as no detailed study exists focused on this species.

### B. Patterns and history of the two watersheds contacts

We found evidence of faunal contacts between the two studied river systems (Fig. 2). These patterns differ remarkably in the hypothesized distribution scenarios. Although in both species, the connection between the Okavango and Cuanza population has been observed in the same location, the prominent difference is represented by the direction of the putative colonization events happening between these two river systems. In *Tilapia sparrmanii*, the colonization most likely happened from the Cuanza to Okavango river system (Fig. 2A), while in *Serranochromis macrocephalus*, the opposite scenario is suggested: there was one colonization event from the Okavango river system to the Cuanza, followed by a potentially bi-directional second colonization event between these two river systems (see Fig. 2B, D). It could be also proposed that the different pattern is caused by missing samples of *Tilapia* from the Rio Cutato (Okavango), as we do not have any *Tilapia* samples from this location and the Cutato population represents the basal-most clade of *S. macrocephalus* tree. However, even not considering the Cutato population, the patterns of the other populations still differ between species. In addition, these two species differ in the age of the lineages – the whole Angolan *S. macrocephalus* lineage is younger (0.13 – 0.23 MY) than the geographically overlapping lineage for *Tilapia sparrmanii* (0.86 – 1.5 MY). However, considering only the subclades with the colonization event between the upper Cuquema (Cuanza) and Cuchi (Okavango) Rivers, both these events of *Tilapia* colonization and the second *Serranochromis* colonization occurred in the end of late Pleistocene (0.048 – 0.085 MYA and 0.039 – 0.069 MY, respectively).

The ichthyofaunal connection between upper Cuanza and upper Okavango has been indirectly reported earlier, mainly based on limited existing faunal records, namely the presence of Okavango–Zambezi species in the upper Cuanza tributaries [28], [32]. This is also why the upper-Cuanza area is considered as a part of a different ecoregion (the “Zambezian Headwaters”), compared to the middle and lower Cuanza (which belongs with the other westward flowing coastal drainages to the ecoregion “Cuanza”; [23]). Unfortunately, detailed study of the Bié plateau and its historical drainage system is still missing. These reported faunal records deal with the interspecific distribution patterns, while the present study enhances current knowledge by providing molecular evidence for the observed faunal connection at the intraspecific level.

It is relatively common for certain fish species to penetrate geographic barriers and disperse more easily, leading them to broader distribution ranges. Many species have a distribution range encompassing all the river basins of the Cunene, Okavango, Zambezi and Congo, indicating a level of permeability of their watersheds [15], [48], [49]. Other rivers (such as the Cuanza) are often not listed in the distribution ranges because of their lower region exploration, but recently some of these widely distributed fishes have been reported from Cuanza as well [50], [51]. The geographic location of the faunal connection of the Okavango and Cuanza River systems was found between the rivers Cuchi (Okavango) and the upper Cuquema (Cuanza) and could be explained by the presence of permeable watershed. This hypothesis [12] assumes that the landscape structure enables episodic and/or prolonged contact between the river systems leading to the dispersal of species, or to limited gene flow between locations for short periods. It is possibly due to the open flat landscape and ephemeral connections happening occasionally, rather than being linked to detectable geological changes [52]. The permeable watershed (Cuchi – Cuquema) located in the headwaters area of the Bié plateau is a relatively flat landscape and appears to enhance the episodic stream contacts, thus potentially having an impact on the distribution patterns of the fishes [52]. Similar (semi)permeable watersheds have been observed in other continents based on studies of several other fish groups (the semipermeability is apparent in the different dispersal rates for fish species differing ecologically, [12], [52]). Freshwater fishes in the landscape of the Guyana Plateau in South America show evidence of repeated faunal contacts between adjacent river systems [52], as well as the observed biogeography patterns of South American gymnotids are also explained by the presence of semipermeable watersheds [53]. In African cichlids, the permeable watersheds due to the episodic faunal connections seem to also be a plausible explanation for some of the species distributions observed (like the observed pattern in *S. macrocephalus*), but more generally there is geological evidence (or at least strong assumption) for many river connections lasting for a prolonged period, such as the Cunene + Okavango [54], Cunene + Zambezi [35], Okavango + Zambezi [15], and the Zambezi and Congo [19].

### C. Effect of the waterfalls and rapids zones to distribution patterns of cichlid fishes

The observed biogeographic pattern in the Angolan lineage of *Serranochromis macrocephalus* could be also partially affected by the presence of a migration barrier, which resulted in a discontinuous distribution pattern within one river system. Fishes from the upstream and downstream parts of the Cuquema River (Cuanza), separated by waterfalls and rapids zone (see Fig. 2D), represent different phylogenetic lineages within the *S. macrocephalus* assemblage; i.e., these two populations were more closely related to the fish coming from more distant localities and/or from different river sub-systems than to each other. Significant effect of waterfalls and rapids zones to the distribution patterns and genetic structure has been previously reported from several studies, for instance in haplochromine cichlids (*Pseudocrenilabrus* and *Serranochromis*) from the Lufubu river [14], in Congolese riverine cichlids of the genera *Teleogramma* and *Lamprologus* [55], in the serrasalmine characid *Colossoma macropomum* within the Madeira and Amazon river systems in South America [5], and in freshwater goby (*Rhinogobius*) populations on the Japanese island of Iriomote [7].

The possible distribution pattern has probably been caused by the colonization scenario from the paleolake centre of origin (recent upper Zambezi) through the Okavango river system to the Cuanza system. Based on the reconstructed phylogenetic tree, the downstream Cuquema populations clustering together with the remote Cuiva River populations (both Cuanza), could be result of the first direct colonization from the Okavango to the Cuanza river basin, while the persistent (or re-opening) connection between Okavango (Cuchi) and upstream Cuquema (Cuanza) allowed another colonization (both directions are equally likely), which happened later. The waterfalls and rapids zone within the Cuquema River could therefore represent the migration barrier contributing to maintain the observed genetic structure within the species *S. macrocephalus*. Due to the limited sample size within this study, other explanations such as random-sampling effect or incomplete lineage sorting cannot be completely excluded from the discussion, although the observed exclusive monophyly of all but one of the populations provides strong evidence that we are observing the existing pattern. In contrast, the same waterfalls and rapids zone barrier within the Cuquema River seems to lack such an effect on the distribution patterns of *Tilapia sparrmanii*, where both upstream and downstream populations are closely related within the studied samples. Unfortunately, too little is so far known about the broader distribution patterns and genetic structure within the species *T. sparrmanii* and its relatives to further interpret this finding.

### D. Phylogeny and biogeography of haplochromine cichlids

Zooming out to a broader level, the phylogenetic and biogeographic patterns within the serranochromine cichlids (i. e. serranochromines *sensu lato* in our study; see Fig. 1) have previously been found as not being completely bound by contemporary river watersheds. It has been shown before that the genetic diversity of serranochromines (*sensu stricto*; subgroup of serranochromines *sensu lato*, see Fig. 1) shows both extremely high divergences within species and localities, as well as almost no divergence between some species and genera across geographically distant localities [13], [21].

The origin of the serranochromine lineage has previously been predicted within the Kalahari Paleolakes region located geographically in the contemporary Okavango - Zambezi watershed [13], although that study was missing most of the basal lineages and was covering only serranochromines (*sensu stricto*). The putative lake radiation was followed by the colonization of surrounding rivers, in the “out of paleolake” scenario [13], [21]. We confirm the pattern of the paleolake origin and subsequent Zambezi-to-Okavango-to-Cuanza colonization direction for the ancestors of the species *Serranochromis macrocephalus*, from our analysis with combined data (this study, [13], [20], [21]). However, it should be again noted that the “out of paleolake” pattern is probably true only for the serranochromines *sensu stricto* (sublineage of serranochromines *sensu lato*; [13]). Contributions of additional sampling covering the underexplored area of the Congo basin and Cuanza river [20] and additional non *S. macrocephalus* material from the Cuanza river (this study), the origin of the whole serranochromine (*sensu lato*) lineage is now hypothesized to be located within the Congo river basin, with significant effect of ichthyofaunal permeability from/to the Cuanza river (see Fig. 1 and [20]). All the basal lineages of serranochromines (*sensu lato*) occur within both the Congo and Cuanza river systems, while the “paleolake” Zambezian lineages are exclusively present more in the crown groups of the overall broader phylogeny (Fig. 1) suggesting the later colonization of this region from the Congo and Cuanza river systems.

## Supporting Information

Figure S1 **Selected specimens of cichlid fishes analyzed in the study.** SL  =  standard length. A-C*) Serranochromis macrocephalus* and *Tilapia sparrmanii* showing evidence of faunal contacts between the Cuanza and Okavango river systems in central Angola. D-F) Other members of serranochromines sensu lato used for larger phylogenetic analysis. Please note that the further taxonomic identification of the ´*Haplochromis* ´ sp. and *Thoracochromis* sp. individuals is still in process.
(TIF)
