# Supplementary figures and images for: Cichlid Fishes in the Angolan Headwaters Region: Molecular Evidence of the Ichthyofaunal Contact between the Cuanza and Okavango-Zambezi Systems
Source: PLoS One. 2013 May 27;8(5):e65047. doi: 10.1371/journal.pone.0065047 (PMC3664563; doi:10.1371/journal.pone.0065047)

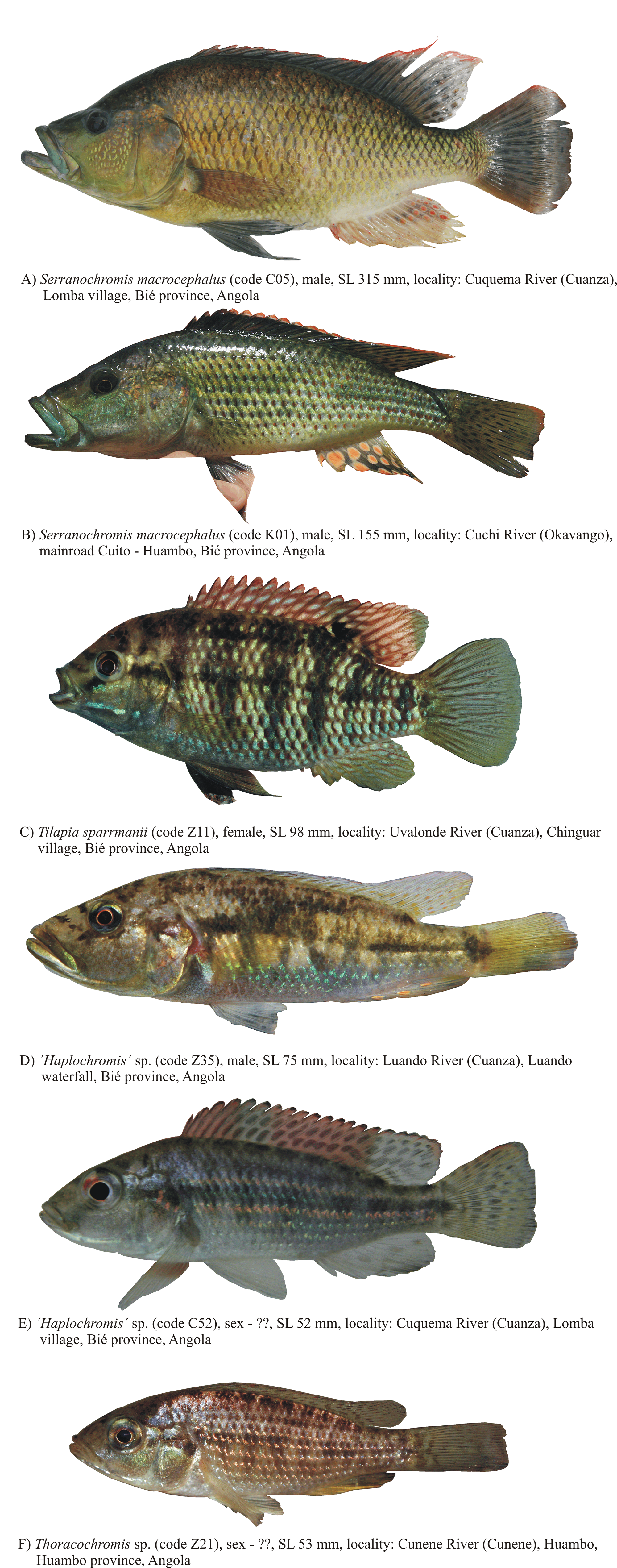

Supplement: Figure S1 — Selected specimens of cichlid fishes analyzed in the study. SL = standard length. A-C) Serranochromis macrocephalus and Tilapia sparrmanii showing evidence of faunal contacts between the Cuanza and Okavango river systems in central Angola. D-F) Other members of serranochromines sensu lato used for larger phylogenetic analysis. Please note that the further taxonomic identification of the ´Haplochromis ´ sp. and Thoracochromis sp. individuals is still in process. (TIF) [file pone.0065047.s001.tif]
